# Reprogramming temozolomide response in glioblastoma through regulated and immunogenic cell death modalities

**DOI:** 10.1038/s41420-026-03151-6

**Published:** 2026-05-26

**Authors:** Tatiana A. Mishchenko, Oluwabukolami J. Olajide, Ekaterina N. Gorshkova, Maria V. Vedunova, Dmitri V. Krysko

**Affiliations:** 1 Institute of Biology and Biomedicine, National Research Lobachevsky State University of Nizhny Novgorod, Nizhny Novgorod, Russian Federation; 2 Cell Death Investigation and Therapy Laboratory, Anatomy and Embryology Unit, Faculty of Medicine and Health Sciences, Department of Human Structure and Repair, Ghent University, Ghent, Belgium; 3 Cancer Research Institute Ghent, Ghent, Belgium

**Keywords:** Cancer therapeutic resistance, Cancer in the nervous system, Cell death and immune response

## Abstract

Temozolomide (TMZ) resistance, driven by genetic, epigenetic, and tumor microenvironment factors, remains a significant barrier in glioblastoma (GBM) therapy. Immunogenic cell death (ICD) modalities (such as necroptosis, ferroptosis, pyroptosis) offer a promising strategy to overcome TMZ resistance by enhancing tumor immunogenicity and reducing adaptive resistance. A strategic shift from conventional TMZ-induced apoptosis to a mixed cell death approach may enhance GBM sensitivity and therapeutic outcomes. However, balancing ICD activation is critical to avoid unintended pro-tumorigenic effects. In this review, we critically evaluate TMZ’s potential to induce multiple regulated cell death modalities, including apoptosis, necroptosis, ferroptosis, pyroptosis and cuproptosis, then map the detailed crosstalk between these regulated cell death pathways and assess their ICD characteristics. From a future perspective, we discuss the challenges associated with TMZ application in GBM therapy and propose novel strategies for developing highly effective GBM therapies and improving long-term treatment outcomes.

## Facts

- TMZ is an alkylating agent used in combination with radiotherapy as the FDA-approved standard of care for GBM; however, its clinical benefits are limited.
- GBM sensitivity to TMZ is complex and depends not only on MGMT expression but also on several epigenetic mechanisms; the list of contributing factors continues to grow.
- Induction of ICD modalities helps transform the GBM microenvironment into an immunologically “hot” state, engaging the immune system and a T-cell immune response for effective tumor eradication.
- A boosted anti-GBM strategy needs mutually beneficial activation of TMZ and ICD modalities, potentially producing mixed-type cell death (MTCD); however, the balance between TMZ and ICD activation should be finely tuned, as excessive or poorly targeted ICD may promote a pro-tumor effect and abrogate the therapeutic efficacy.

## Open questions

- What ICD modality makes an ideal combination with TMZ for effective GBM treatment, or should a strategy for triggering mixed-type cell death be tailored through a personalized approach?
- How can TMZ be safely combined with an ICD inducer without exacerbating neurotoxicity? Specifically, what should the step-by-step strategy be for choosing the optimal pharmacological parameters—such as dosing, timing, and sequencing— to achieve a synergistic anti-tumor effect while maintaining a therapeutic window and preventing off-target toxicity?

## Introduction

Gliomas are the most prevalent type of central nervous system cancers, accounting for about half of all brain tumors. Among these cancers, glioblastoma multiforme (GBM), classified as a World Health Organization (WHO) grade IV glioma, stands out as the most aggressive and deadly form (https://tumourclassification.iarc.who.int) (Box 1). GBM constitutes about 80% of the cancers affecting the central nervous system, which makes it the most prevalent brain cancer [1, 2]. This malignant primary brain tumor has a very poor prognosis, with a median survival of about 15 months [3]. However, 3–5% of patients survive more than 36 months, and 0.71% survive more than 120 months [4].

The current treatment of GBM requires a multifaceted approach and can be challenging mostly due to its varying nature, location beyond the blood-brain barrier, high heterogeneity, and hostility of the GBM microenvironment to immune cells. The first step in therapy is often maximal safe surgical resection, the extent of which depends on the site and importance of the affected brain area. This is followed by radiotherapy (RT) and concurrent chemotherapy [5, 6]. While the standard approach follows this conventional sequence, emerging evidence suggests that preoperative (neoadjuvant) RT is gaining interest [7]. This approach is used in brain metastases to sterilize the tumor bed and improve local control [8], but it has not been explored sufficiently in primary GBM. Recent preclinical evidence demonstrates that neoadjuvant RT significantly prolongs survival and modulates the tumor immune microenvironment compared to adjuvant RT in a GBM model [7]. The proof-of-concept of neoadjuvant RT has prompted early-phase clinical trials, including the POBIG trial designed to assess the safety and feasibility of single-fraction preoperative RT (ClinicalTrials.gov: NCT03582514) [9] and a preoperative radiosurgical boost study (ClinicalTrials.gov: NCT05030298). Importantly, this strategy remains experimental for primary GBM and is not part of current standard-of-care guidelines.

Chemotherapy is essential for patients with GBM to deter postoperative recurrence. However, despite the availability of various chemotherapeutic drugs, only a handful of them are commonly used clinically for glioma treatment [10, 11].

Temozolomide (3-methyl-4-oxoimidazo[5,1-d] [1–3, 5] tetrazine-8-carboxamide, TMZ), also known as Temodal, is a small lipophilic molecule used in the treatment of certain types of brain tumors. TMZ was first tested in a phase I clinical trial for gliomas in 1987, followed by approval from the Food and Drug Administration in 1999 for recurrent GBM and anaplastic astrocytoma. It was approved in 2005 as a first-line chemotherapeutic agent for newly diagnosed GBM [5, 12]. Stupp and colleagues demonstrated that radiotherapy combined with TMZ significantly improved the overall survival of patients with GBM [5] (Box 2). This paradigm shift means that TMZ should be used as a part of combination therapy for meaningful clinical efficacy [13]. The proposed strategy quickly gained global acceptance and implementation. Since 2005, TMZ combined with radiotherapy has become an essential component of standard-of-care therapy for GBM, though its therapeutic benefit is influenced by genetic factors such as methylguanine-DNA methyltransferase (MGMT) mutation and functional DNA mismatch repair (MMR) [14].

The persistent challenge of GBM resistance to conventional therapies, including TMZ, underscores the urgent need to reevaluate both current and new combinative approaches to enhance therapeutic efficacy. A wealth of compelling evidence from a wide range of basic and clinical studies conducted over the past few decades has underscored the importance of engaging the immune system and a T-cell immune response for effective tumor eradication. This quickly led to interest in immunotherapy as a promising solution to the challenges posed by aggressive GBM growth, metastasis and secondary tumor development. Several immunotherapeutic approaches are currently adapted for anti-cancer treatment. These include immune checkpoint inhibitors, chimeric antigen receptor T cell therapy, gene-mediated cytotoxic immunotherapy, oncolytic virus-based therapy, and therapeutic anti-tumor vaccination (dendritic cell-based vaccines and DNA/RNA vaccines). However, immunotherapy of GBM in clinical settings has not been sufficiently effective. The main hurdle in GBM immunotherapy accompanies with the tumor microenvironment, which impedes T-cell infiltration and activation [10]. In this context, approaches based on the induction of immunogenic cell death (ICD, Box 3) hold significant potential to transform the tumor microenvironment into an immunologically “hot” state and may act as a powerful tool to enhance therapeutic outcomes [10, 15–18].

From that perspective, we critically evaluate TMZ’s potential within contemporary combination treatments. We focus on how TMZ acts as a single agent in experimental models, and also in combination with therapeutic agents to engage multiple regulated cell death modalities: apoptosis, necroptosis, ferroptosis, pyroptosis, and the recently described cuproptosis. Then we map in detail the crosstalk between these regulated cell death pathways and assess their ICD characteristics. Finally, we address the key challenges in using TMZ in GBM therapy and propose novel strategies for developing effective GBM therapies.

Box 1 Primary and secondary GBM: which is which? GBM is classified as primary or secondary based on clinical manifestation. Primary GBMs account for 80% of cases and predominantly affect older individuals, with an average onset age of 64 years [2, 3]. The features of primary GBMs include significant genetic alterations, such as mutations and amplification in the epidermal growth factor receptor (EGFR) gene, overexpression of mouse double minute 2 (MDM2), deletion of p16, telomerase reverse transcriptase (TERT) promoter mutation, and loss of heterozygosity of chromosome 10q, which affects the phosphatase and tensin homolog (PTEN) [128].

The secondary GBMs, on the other hand, may arise from lower-grade astrocytomas or oligodendrogliomas, which are characterized by overexpression of platelet-derived growth factor A (PDGFA) and platelet-derived growth factor receptor alpha (PDGFRα), retinoblastoma, loss of heterozygosity of 19q, and mutations in isocitrate dehydrogenase 1 and 2 (IDH1/2), tumor protein 53 (TP53), and α-thalassemia/mental retardation syndrome X-linked (αTRX) [128]. This class of GBM is seen in younger patients with an average age of 45 years [129]. The importance of genetic profiling of GBM for tailoring treatment strategies cannot be overlooked. For instance, the status of the TP53 gene can influence the efficacy of the chemotherapy. Tumors that have functional TP53 are more likely to respond to chemotherapy such as TMZ compared to tumors with mutated TP53, which might be resistant [130]. Secondary GBMs may have more or less sensitive to chemotherapy than primary GBMs, which typically have a more aggressive profile [128, 131].

Box 2 Standard of care for the treatment of GBM according to the Stupp protocol Stupp and colleagues performed a randomized study on 573 patients in 85 centers. The median age of the patients was 56 years, and 84% of them had undergone debulking surgery. This work illustrated that combining TMZ with RT notably extends survival rates in individuals diagnosed with GBM, resulting in a median survival increase from 12.1 to 14.6 months and increasing the percentage of patients surviving for two years from 10% to 26%. Currently, the Stupp protocol involves surgical removal of the tumor to the fullest extent possible, followed by targeted RT to the affected area (60 Gray [Gy] at 2.0 Gy per day for 42 days). Simultaneously, patients receive daily TMZ chemotherapy doses of 75 mg/m^2^. Following completion of the RT—TMZ chemotherapy, patients proceed to six cycles of adjuvant TMZ maintenance therapy. During this phase, patients receive higher doses of TMZ (150—200 mg/m^2^) for five consecutive days every 28 days [5, 6].

Box 3 An overview of immunogenic cancer cell death modalities The term ***immunogenic cell death (ICD)*** encompasses several forms of regulated cell death that can be triggered by current antitumor treatments [17, 109]. The main feature of ICD is gradual release of signaling molecules known as damage-associated molecular patterns (DAMPs), which are normally hidden from the immune system but are released extracellularly during cell death. The released DAMPs attract antigen-presenting cells (*i.e*., dendritic cells; DCs) and are responsible for the adjuvanticity of dying cells. Chemokines and cytokines released from the dying cancer cells can also contribute to their immunogenicity [132]. Moreover, cancer cells undergoing ICD are antigenic due to their tumor-associated antigens and tumor-specific antigens. The close interplay between the adjuvant and antigenic properties of ICD culminates in the presentation of cancer antigens on major histocompatibility complex class I (MHC I) molecules to CD8^+^ T cells. This process ultimately leads to effective tumor eradication and the generation of long-lasting immunological memory, underscoring its therapeutic potential.

The type of induced cell death can be confirmed in vitro [107, 133] and validated In vivo in syngeneic immunocompetent animals. In vivo analysis includes prophylactic heterotopic or orthotopic vaccination with ICD-treated tumor cells killed in vitro, followed by a rechallenge with the same type of viable cancer cells [106, 107].

**The key ICD modalities and their specific characteristics**

***Immunogenic apoptosis*** has similar morphological features to conventional apoptosis and is associated with chromatin condensation, chromosomal DNA cleavage into internucleosomal fragments, membrane blebbing, and formation of apoptotic bodies. In the biochemical cascade, immunogenic apoptosis in parallel with caspase activation often requires the induction of endoplasmic reticulum stress and reactive oxygen species [109, 110]. This cell death process can be blocked by the pan-caspase inhibitor zVAD-fmk [111].

***Necroptosis*** was discovered as a caspase-independent programmed form of necrotic cell death [112]. It can be induced by ligation of TNF receptor family proteins (including TNFR, FAS, TRAILR and DR6) when caspase-8 activity is blocked. Necroptosis is mainly mediated by activation of receptor-interacting protein kinase 1 (RIPK1), receptor-interacting protein kinase 3 (RIPK3) and mixed lineage kinase (MLKL) domain [113, 114]. It can be blocked by inhibitors of RIPK1 (*e.g*., Nec-1 s), RIPK3 (*e.g*., GSK'872), and MLKL (*e.g*., necrosulfonamide for human cells only) [115]. Necroptosis is also considered a type of ICD [116, 117].

***Ferroptosis*** is driven by the iron-dependent accumulation of lipid peroxides, particularly within phospholipid membranes, leading to cell membrane damage and death. It is characterized by the depletion of intracellular glutathione and inactivation of glutathione peroxidase 4 (GPX4), which protects cells from oxidative damage [118, 119]. Unlike apoptosis and necroptosis, ferroptosis results from an imbalance between oxidants and antioxidants causing unchecked peroxidation of polyunsaturated fatty acids. Among the several ferroptosis inhibitors are ferrostatin-1, liproxstatin-1, α-tocopherol, zileuton, FSP1, and CoQ10 [115]. The induction of ferroptosis in cancer can be immunogenic [120, 121] and represents a plausible strategy to overcome resistance to other cell death modalities [122].

***Pyroptosis*** is an inflammatory form of regulated cell death [123]. Upon pyroptosis induction, the inflammasome, a complex of several proteins (such as NLRP1, NLRP3, NLRP4, IRAF, and AIM2), drive the activation of caspase-1 or caspase-11, −4, and −5, resulting in the cleavage of gasdermin D (GSDMD). Following apoptosis-like DNA fragmentation, chromatin condensation, and cellular swelling, the cleaved GSDMD’s N-terminal fragment is embedded into the cell membrane and causes necrosis-like morphological changes such as plasma membrane permeabilization. The eventual loss of cell integrity accompanied by the release of cellular contents, including pro-inflammatory cytokines (*i.e*., IL-1β and IL-18), leads to a severe inflammatory response and cell death [124]. A list of pyroptosis inhibitors affecting NLPR3 or Caspase 1 has been described [115].

***Cuproptosis*** is a recently discovered copper-dependent form of regulated cell death [80] associated with mitochondrial metabolism and protein lipoylation. It is triggered by accumulation of excess cupric ions in mitochondria, where it is reduced to the cuprous state (Сu^+^) by ferredoxin (FDX1). This process leads to either depletion of iron–sulfur (Fe–S) cluster proteins or direct interaction with lipoylated enzymes (*i.e*., dihydrolipoamide S-acetyltransferase (DLAT) and dihydrolipoamide S-succinyltransferase (DLST)) in the tricarboxylic acid cycle. These events culminate in proteotoxic stress, oxidative damage, and ultimately, cell death [80, 125]. Cuproptosis can be induced by increasing intracellular copper levels using small-molecule copper ionophores, including elesclomol [126], disulfiram (DSF, an aldehyde dehydrogenase inhibitor) [125] and NSC319726 [127]. The uniqueness of cuproptosis is characterized by its insensitivity to inhibitors of other regulated cell death pathways, such as antiapoptotic zVAD-fmk, antinecroptotic necrostatin-1, antiferroptotic ferrostatin-1 or N-acetyl cysteine, a suppressor of oxidative stress. Beyond the established ICD modalities, cuproptosis is considered an alternative form of regulated cell death with a promising immunogenic potential, the role of which in tumor immunotherapy requires further investigation [81, 82].

## Mechanism of TMZ action and its Achilles’ heel in GBM therapy

TMZ acts as an alkylating agent and serves as a prodrug for 3-methyl-(triazen-1-yl) imidazole-4-carboxamide (MTIC) [19]. TMZ stands out from conventional alkylating agents such as carmustine, procarbazine and lomustine [19, 20], which have high general toxicity and limited efficacy in prolonging patient survival. In contrast, TMZ solves these issues by bypassing metabolism involving cytochrome P450 enzymes and kidneys. Also, as extensively documented, its side effects are typically predictable and reversible, ranging from mild to moderate severity [21]. Of note, TMZ is suitable for treating GBM because it is one of the few drugs that can cross the blood-brain barrier.

**Fig. 1:**
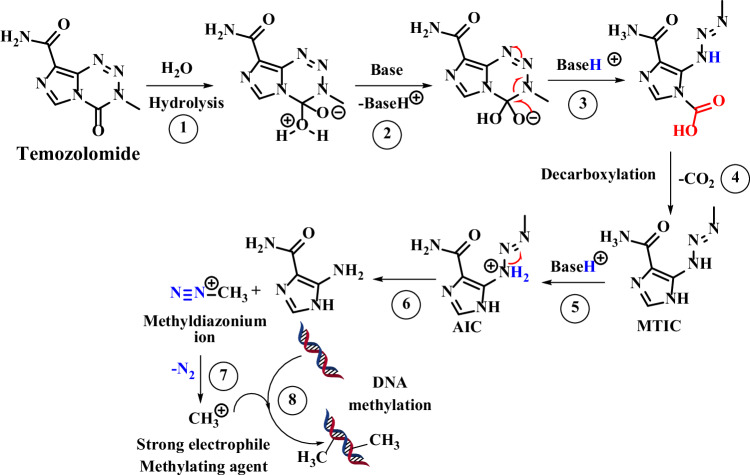
An overview of conversion of TMZ to MTIC. **1** TMZ undergoes spontaneous hydrolysis, particularly under physiological conditions (pH ~ 7.4). **2, 3** The following nucleophilic attack by a base (water molecule) generates a product with an open-ring structure. **4** The intermediate structure loses a molecule of CO_2_, leading to the formation of MTIC. **5, 6** MTIC undergoes cleavage to form the methyldiazonium ion, and 5-aminoimidazole-4-carboxamide (AIC) is produced as a byproduct. **7, 8** The methyl diazonium ion acts as a methylating agent, transferring the methyl group to various nucleophilic sites in DNA, such as guanine residues. Alkylation is the key cytotoxic action of TMZ, leading to DNA damage and triggering apoptosis in cancer cells.

TMZ is more stable in acidic media (pH ≤ 5.0) than in basic media (pH ≥ 7.0) due to the protonated form that minimizes catalysis [20]. Consequently, it is totally and rapidly hydrolyzed to MTIC within two hours (Fig. 1) and is about 100% bioavailable, but has a half-life of 1.8 h and narrow brain distribution (17.8%), necessitating repeated dosing [20].

**Fig. 2:**
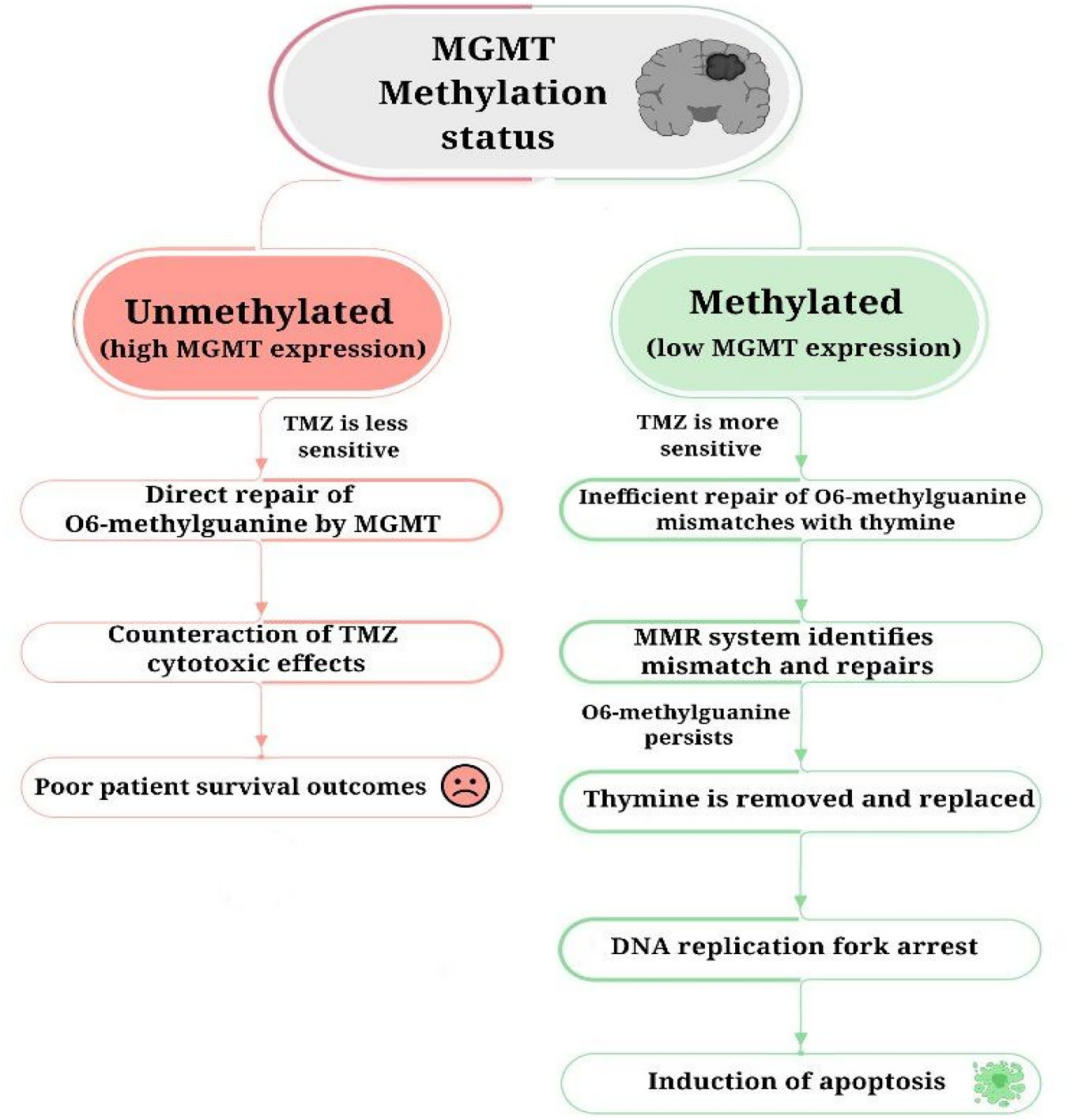
Schematic representation of the impact of MGMT methylation status on TMZ-induced apoptosis. The flowchart illustrates the decisive and contrasting impact of MGMT methylation status on the effectiveness of TMZ treatment and patient survival outcomes in two scenarios. In tumors with unmethylated MGMT (high MGMT expression), direct repair of O6-methylguanine lesions leads to resistance against TMZ’s cytotoxic effects, resulting in poorer survival outcomes. Conversely, in tumors with methylated MGMT (low MGMT expression), inefficient repair of O6-methylguanine lesions leads to persistent DNA mismatches, triggering the mismatch repair (MMR) system. This ultimately leads to DNA replication fork arrest and induction of apoptosis, enhancing TMZ sensitivity and potentially improving patient survival.

Despite its widespread incorporation into multimodal GBM treatment regimens, its clinical benefits are limited. Not all GBM patients respond to TMZ, and those who do eventually develop resistance and experience tumor relapse [22, 23]. The cause and mechanisms of developing resistance to TMZ in GBM cells are not fully understood. To date, the methylation status of the MGMT promoter is a well-characterized player determining GBM sensitivity to TMZ (Fig. 1) [24, 25]. MGMT is an enzyme that directly repairs O6-methylguanine adducts. Its high expression levels counteract the cytotoxic effects of TMZ and are associated with poor patient survival [26] (Fig. 2). Consequently, 50-55% of patients undergoing treatment may not respond to TMZ because of MGMT-mediated repair mechanisms [27]. However, TMZ remains a standard treatment in clinical practice despite these limitations [28].

The effectiveness of TMZ in combating tumors also depends on the MMR system of tumor cells. When O6-methylguanine mis-pairs with thymine, the MMR machinery identifies the mismatch, and the mispaired thymine is replaced with yet another thymine during repair synthesis, initiating a cycle of DNA repair that consumes energy [29]. However, the methylated guanine, which MMR cannot correct, remains on the opposite DNA strand. The persistence of methylated guanine can lead to replication fork arrest and possibly formation of DNA double-strand breaks, ultimately triggering apoptosis (Fig. 2) [30].

However, MGMT is not the only factor influencing TMZ’s effectiveness against GBM. For instance, when GBM cells (*e.g*., human U-87 and LN229 cell lines) were exposed to TMZ, miR-1268a was identified as a significant factor in regulating resistance to TMZ by its ability to target ABCC1 to modulate TMZ sensitivity [31]. In that study, it was observed that miR-1268a was downregulated in the abovementioned TMZ-treated GBM cells, leading to upregulation of ABCC1, a membrane transporter protein known for expelling chemotherapy drugs from the cell and contributing to drug resistance. Moreover, KDM5A (a histone lysine demethylase gene) was identified as an important gene in controlling TMZ resistance [32]. Enhanced resistance to TMZ by increased expression of this gene has been observed, as well as increased sensitivity due to reduced expression. Crucially, these elements play a role in TMZ resistance in both MGMT-positive and MGMT-negative human GBM, revealing a resistance landscape that is more complex than previously understood. Several experimental data have demonstrated that TMZ-induced resistance can emerge in MGMT-negative cells through alterations in other DNA repair pathways, as well as changes in the transcriptome, proteome, and metabolome. Such alterations undermine TMZ’s efficacy and contribute to GBM recurrence after treatment. Additionally, while MGMT promoter methylation is a well-established prognostic marker (Fig. 1), its predictive value for TMZ responsiveness is not consistently reproducible across all GBM subtypes. This situation has led researchers to explore alternative biomarkers and resistance mechanisms, highlighting the role of epigenetic modifications in shaping cellular responses to TMZ [33, 34].

Overall, while MGMT remains a critical player in TMZ resistance, it is clear that numerous MGMT-independent mechanisms also play a significant role in TMZ resistance mechanisms. This growing body of research highlights the complexity of GBM resistance and strongly indicates the need for novel therapies of higher efficacy.

## Crosstalk between TMZ and ICD modalities in GBM therapy

### Temozolomide and apoptosis

**Fig. 3:**
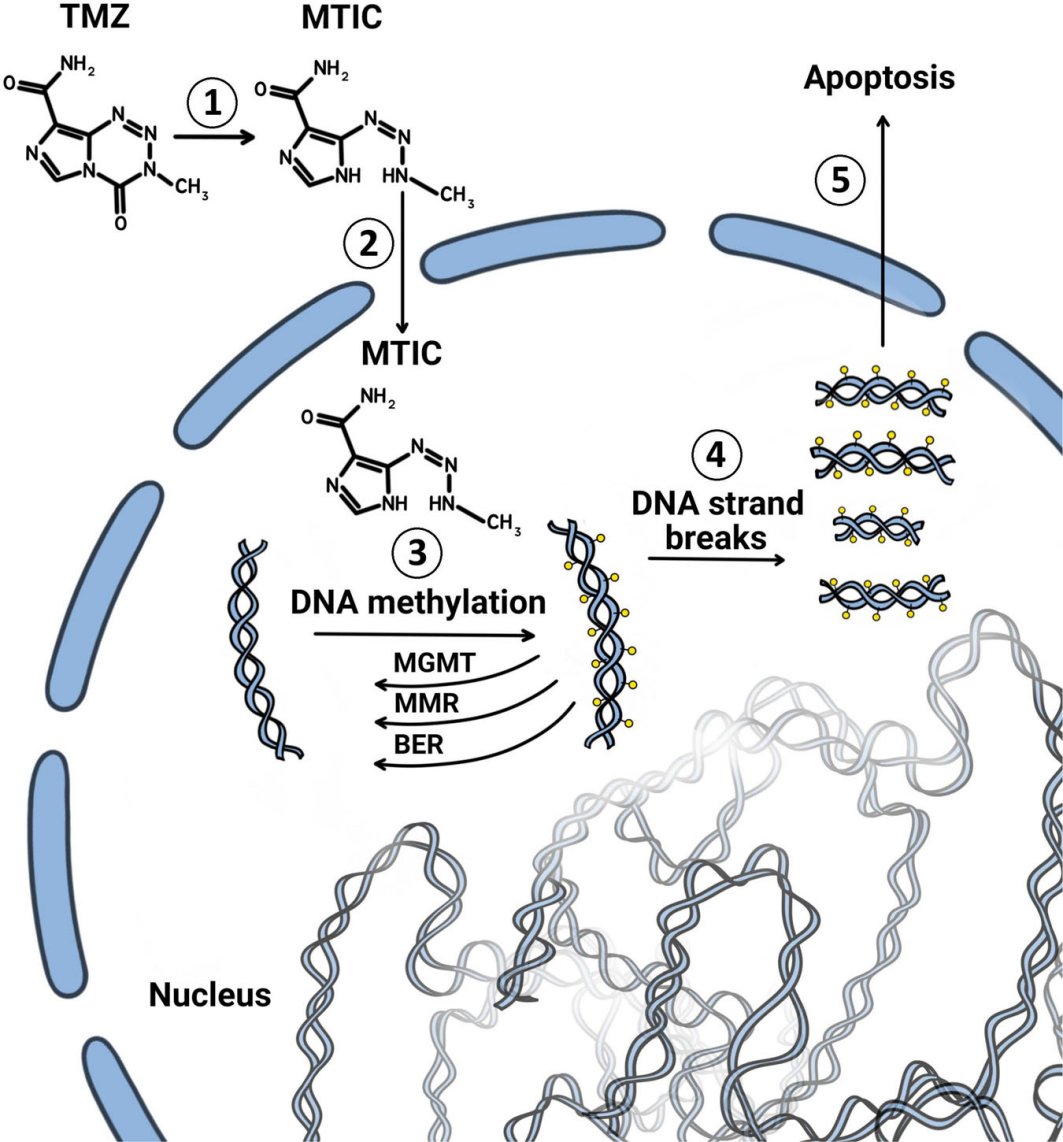
Mechanism of action of TMZ-induced apoptosis. **1** TMZ is hydrolyzed to MTIC in the body at a pH of 7.4. **2** MTIC enters the cell nucleus. **3** MTIC methylates the DNA at the N7 position of guanine (70%); the O6 position of guanine (6%) and at the N3 position of adenine (9-20%). The N7 and N3 lesions are readily repaired by the base excision repair (BER) pathway, minimizing their impact. **4** The antitumor activity of TMZ primarily targets the O6-methyl-guanine and the effectiveness of this depends on a functional status of DNA-mismatch repair (MMR) in the tumor cells and on MGMT expression. O6-methylguanine mispairs with thymine, which is recognized by the MMR machinery. The mispaired thymine is replaced with yet another thymine during repair synthesis, leading to futile, energy-consuming cycles of DNA repair. **5** The methylated guanine, which cannot be repaired by MMR, persists on the opposite strand, leading to replication fork arrest and presumably to DNA double-stranded breaks and eventual apoptosis.

Apoptosis (Box 2) represents the primary and well-characterized mechanism through which TMZ exerts its effects [35, 36]. In brief, in aqueous solutions such as the blood, TMZ is spontaneously hydrolyzed to MTIC **(**Fig. 2**)**, which exerts the antitumor activity by breaking down into 5-aminoimidazole-4-carboxamide (AIC) and the active compound, methyldiazonium cation, which subsequently alkylates the DNA (Fig. 1). This methyldiazonium cation primarily methylates guanine residues in DNA [37, 38]. DNA methylation occurs mostly at the N7 position of guanine (70%), the O6 position of guanine (6%), and the N3 position of adenine (9%) [38]. Formation of O6-methylguanine is believed to initiate a sequence of futile DNA mismatch-repair events that can induce apoptosis **(**Fig. 3**)**.

Of note, the N3-methyladenine and N7- methylguanine lesions are easily repaired by the base excision repair; therefore, it is assumed that they contribute much less to the apoptotic effects [39, 40].

Although O6-methylguanine lesions are less abundant than N3-methyladenine and N7-methylguanine lesions, they are more toxic. Therefore, it has been reported that the antitumoral activity of TMZ primarily targets O6-methylguanine [41]. This activity strongly depends on functional MMR in GBM cells and on MGMT expression.

Beyond its role in inducing DNA damage, the effectiveness of TMZ critically depends on intact mitochondrial apoptotic signaling [42, 43]. Although cytochrome С, a fundamental component of the respiratory chain essential in mitochondria-induced apoptosis, does not cause gliomagenesis, impairment of its release from mitochondria into the cytoplasm is a known feature of GBM progression and contributes to intrinsic apoptosis resistance. [44, 45]. Mitochondrial membrane permeability GBM cells is frequently altered, limiting cytochrome C-mediated caspase activation even in the presence of extensive TMZ-induced genotoxic stress [43, 44]. Moreover, elevated cytochrome С oxidase activity has been implicated in metabolic adaptations contributing to TMZ resistance in established human GBM cell lines (*e.g*., U87, U251) and patient-derived GBM cultures, enhancing oxidative phosphorylation and ATP production, which are associated with treatment resistance [42, 46]. Retrospective clinical data also suggest a correlation between higher tumor cytochrome C oxidase activity and worse outcomes in GBM patients, although its prospective validation as a prognostic biomarker remains limited [47, 48]. These mitochondrial and metabolic adaptations actively suppress apoptotic execution downstream of DNA damage, representing a critical but often underappreciated weakness in TMZ-based therapy.

### Differential sensitivity to apoptosis of TMZ-treated GBM cell lines

GBM cell lines exhibit varying degrees of sensitivity to TMZ, reflecting differences in their response to treatment. It has been shown that TMZ treatment in a three-dimensional spheroid culture model affects GBM cell lines differently. Specifically, U-87 MG, a human GBM cell line with a functional p53 gene, exhibited a classic pattern of apoptosis and senescence within 48 hours [49]. In contrast, human GaMG cells exhibited greater resistance to TMZ, likely due to deficient p53 function, resulting in reduced sensitivity to TMZ-induced apoptosis. Of note, another study investigating the impact of methylating agents such as N-methyl-N′-nitro-N-nitrosoguanidine and TMZ, on malignant human GBM cell lines, including U-87 MG (p53 wild-type), U-138 MG (p53 mutant), LN-308 (p53 mutant), MO59K DNA-PK wild-type, and MO59J DNA-PK mutant glioma cells, found that exposure to these agents induced apoptosis [50]. Notably, U-87 MG (p53 wild-type), exhibited greater sensitivity to apoptosis induction than cells with mutated p53. Interestingly, another study on survival and cell death mechanisms in human LN229 and U-87 MG GBM cell lines indicated that disrupting autophagy following TMZ treatment increases tumor cell susceptibility to TMZ-induced DNA damage, ultimately leading to apoptosis [51]. These studies show that TMZ sensitivity in human GBM cell lines depends on several factors. Among them are p53 status and cellular mechanisms such as autophagy, which can shift the balance between apoptosis and senescence during TMZ treatment.

### How to enhance apoptotic sensitivity to TMZ?

It has been shown that combining TMZ with other therapies offers a promising approach to enhancing apoptotic sensitivity and overcoming resistance in GBM treatment. In this regard, it has been reported that the anticancer effects of SB747651A, a multi-target small-molecule inhibitor of protein kinases, specifically the cyclin-dependent kinase 9, in combination with TMZ, reduced cell proliferation, spheroid formation, migration and chemoresistance in three well-characterized patient-derived GBM spheroid cultures [52]. The authors demonstrated an increase in apoptosis, particularly in patient-derived primary GBM spheroid cultures (T78 and T111) with methylated MGMT promoters. In contrast, the patient-derived primary glioblastoma T86 spheroid culture, which has an unmethylated MGMT promoter, exhibited strong resistance to both TMZ and the targeted therapy. Recently, in vitro study of patient-derived GBM spheroids and in silico studies evaluated the effectiveness of combining TMZ and doxorubicin in treating GBM [53]. The authors demonstrated that this combined chemotherapeutic regimen resulted in a greater induction of apoptosis.

Interestingly, TMZ-based sonodynamic therapy (SDT) has been shown to be more effective in inducing cell death in human LN229 and mouse GL261 GBM cells compared to TMZ alone [22]. The authors demonstrated that TMZ-based SDT promoted ICD characterized by emission of DAMPs, leading to activation of bone marrow-derived DCs. In vivo, TMZ and SDT synergized to create a more favorable GBM microenvironment for immune cells and to elicit robust anti-tumor immune responses. Additionally, the authors demonstrated that using an engineered nanoliposome vector for TMZ improved SDT tumor targeting, suggesting safer clinical application for the combination therapy of TMZ and SDT [22].

**Fig. 4:**
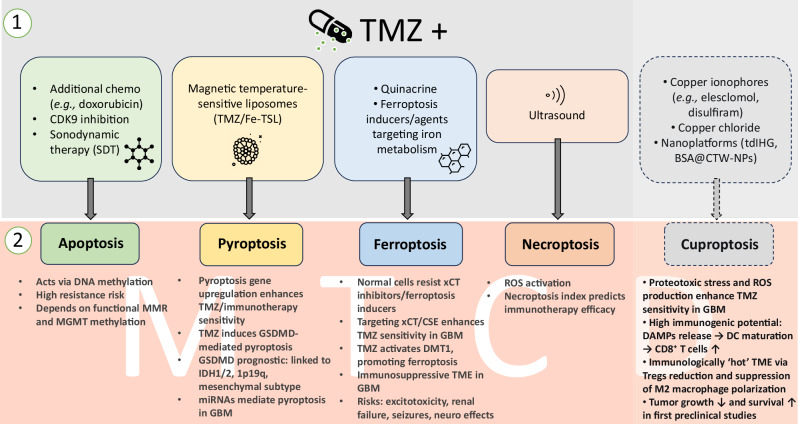
A therapeutic approach integrating temozolomide (TMZ) with immunogenic cell death (ICD)-inducing agents to trigger mixed-type cell death (MTCD) aimed at improving outcomes of glioblastoma (GBM) treatment. **1** A synergistic strategy simultaneously activates TMZ’s canonical apoptotic pathway in GBM cells and engages ICD-linked pathways (e.g., pyroptosis, ferroptosis, necroptosis), and newly emerged cuproptosis with prospective immunogenic potential, enabling dual induction of regulated cell death. **2** This therapeutic integration exploits the distinct immunogenic properties of ICD and their synergistic interplay to remodel the immunologically inert tumor microenvironment, sensitize neoplastic cell populations to cytotoxic therapy, and mechanistically elicit robust antitumor immunity *via* MTCD induction.

In conclusion, TMZ has demonstrated significant therapeutic potential against GBM, primarily through its ability to induce apoptosis *via* DNA methylation, particularly at O6-guanine sites (Fig. 3). This methylation triggers a cascade of DNA mismatch-repair events leading to apoptosis when repair mechanisms, such as MGMT, are methylated. While TMZ is primarily known to induce apoptosis through DNA methylation at O6-guanine sites, the exact apoptotic mechanisms remain complex and context-dependent and often associated with therapy resistance. Therefore, one possible approach to overcoming such resistance mechanisms is the use of combination therapies to enhance TMZ’s antitumor effects. Approaches such as SDT, autophagy inhibition, or combining TMZ with agents like doxorubicin represent promising strategies to increase GBM sensitivity to TMZ **(**Fig. 4**)**. Importantly, these combined therapies not only enhance TMZ’s apoptotic action but also trigger ICD (Box 3), offering multifaceted approaches to overcoming resistance in GBM treatment.

## TMZ and necroptosis

A growing body of evidence supports a functional interplay between TMZ and necroptosis (Box 3), a regulated form of necrotic cell death, suggesting its potential role in overcoming drug resistance in GBM therapy (Fig. 4). TMZ combined with ultrasound increases ROS production and the levels of phosphorylated RIPK1, RIPK3 and MLKL, and thereby induces necroptosis in human GBM cell lines (U-87, U-251 and LN229) and primary cells isolated from GBM patients [54]. Furthermore, recent studies have revealed that necroptosis can be used as a therapeutic target and prognostic indicator in gliomas, especially low-grade glioma and GBM [55]. The necroptosis index in glioma patients predicts responses to immunotherapy and targeted treatment [56].

Although necroptosis shows promise as an alternative cell death pathway for GBM treatment, its complex regulatory mechanisms require further investigation to develop and optimize effective therapeutic strategies. To date, there is no clinical or experimental evidence that GBM cells acquire resistance specifically to necroptotic stimuli. The primary challenge appears to be intrinsic GBM resistance, driven mainly by epigenetic silencing of key players of the necroptotic pathway, rather than therapy-induced acquired resistance. For example, 2-hydroxyglutarate produced by IDH1 mutant GBM binds to DNA methyltransferase 1 and induces hypermethylation of the RIPK3 promoter which suppresses its expression thereby rendering cells refractory to necroptotic stimuli [57]. This suggests that a subset of GBMs may be inherently resistant to necroptosis. Therefore, future strategies should seek to overcome this innate resistance to unlock the pathway’s potential. Combinatorial approaches, such as pairing TMZ with epigenetic modulators to re-sensitize cells, or with agents that induce lethal ROS production, could be crucial to enforce necroptotic cell death and achieve durable therapeutic responses.

## TMZ and ferroptosis

### Ferroptosis: an efficient strategy to treat GBM

Emerging evidence suggests that TMZ can induce ferroptosis (Box 3, Fig. 4). Long-lasting TMZ treatment induces a quiescent cell state in GBM characterized by altered mitochondrial oxidative phosphorylation [58], leading to stronger tumor aggressiveness and dismal prognosis [59]. Using genetically engineered mouse glioma models (with deletion of p53 alone or with constitutively active Notch signaling [N1IC] and patient-derived cell lines), researchers have shown that astrocyte-like cells in GBM modulates mitochondrial lipid peroxidation at complex I. This process directly impacts the response to GPX4 inhibitors and susceptibility to ferroptosis [60]. Studies have shown that TMZ treatment alone leads to the accumulation of ROS in human glioblastoma TG905 cells and targets the divalent metal transporter DMT1, which is responsible for iron homeostasis [61]. Subsequent DMT1 upregulation induces ferroptosis. Moreover, it has been shown that the administration of TMZ combined with the autophagy inhibitor quinacrine increases susceptibility to ferroptosis in human GBM stem-like cells, which are primarily responsible for GBM aggressiveness [62].

Interestingly, several research groups together suggest combining TMZ with ferroptosis inducers or agents targeting iron metabolism to enhance TMZ efficiency and overcome GBM cells’ resistance to TMZ. For example, studies using human GBM cell lines (A172, U-87 MG and T98G) have shown that TMZ resistance involves TMZ-induced xCT upregulation and cystathionine γ-lyase activation by increasing the expression of NF-E2-related factor 2 (Nrf2) and activating transcription factor 4 (ATF4), as well as the transsulfuration pathway [63]. This effect can be mitigated by erastin, which targets xCT and significantly inhibits CTH activity, leading to ROS accumulation and increased TMZ cytotoxicity.

Further research into the role of NRF2 in GBM cells and its connection to ferroptosis revealed that human glioblastoma T98G cells, which have high NRF2 expression, are more resistant to TMZ [64]. However, silencing NRF2 rendered these cells more sensitive to TMZ, highlighting the role of NRF2 in ferroptosis induction through GSH production. Elevated levels of NRF2 make tumors more resistant to chemotherapy, but increases their vulnerability to ferroptosis when GSH production is inhibited [64]. Another study employing various xCT inhibitors and TMZ revealed that the level of xCT expression dictates the sensitivity of glioma cells to TMZ in humans, but not in rodents [65]. Strong xCT expression prompts human glioma cells to respond to combined TMZ treatment with erastin, but not with sorafenib [65]. Interestingly, the authors revealed that healthy astrocytes and neurons are less vulnerable to xCT inhibitors and ferroptosis inducers than glioma cells, which could be a way to avoid the development of cytotoxic side effects in healthy brain cells [65].

In the context of modulating GPX4 expression, RSL3 together with TMZ significantly increase cell death in GBM cell lines (LN18, LN229, and primary GBM cell lines) [23]. The RSL3 contribution to TMZ sensitization is reflected in morphological alterations, specifically the rupture of mitochondria and other organelles, downregulating the expression of genes involved in the cell cycle and responsible for epithelial-to-mesenchymal transition. Interestingly, the reduced invasive mobility of GBM cells occurs in both mutant and wild-type IDH1 cells [23]. Furthermore, it has been shown that application of ALZ003, a structural analogue of the biologically active iron chelator curcumin, impairs androgen receptor-regulated GPX4 expression, leading to lipid peroxidation and abundant ROS accumulation in human glioblastoma U87MG cells, dramatically decreasing the size of TMZ–resistant GBM tumors In vivo [66].

These studies indicate that combining TMZ with ferroptosis inducers or agents targeting iron metabolism is a promising strategy to overcome TMZ resistance and enhance its efficacy in treating GBM. This approach leverages vulnerabilities in GBM cells, such as xCT expression and GPX4 activity, while sparing healthy brain cells, paving the way for more effective and selective therapies.

### The other side of the coin: ferroptosis and TMZ in GBM progression

Several studies suggest that ferroptosis may play a role in the development of glioma cell resistance to TMZ. One proposed mechanism involves the correlation between ferroptosis-related gene expression levels, glioma grade, and tumor resistance to TMZ. Using human glioma cell lines (A172, H4, U87, U251 and U118) and human glioma primary cells, it has been shown in vitro that silencing Gpx4 inhibits the proliferation and migration of glioma cells and leads to apoptosis [67]. It has also been demonstrated that ATF4, which regulates the transcription of the glutamate antiporter xCT/SCL7A11, can enhance cellular resistance to ferroptosis and cytotoxic stress, thereby facilitating tumor progression and reducing the efficacy of TMZ [68].

On the other hand, recent detailed bioinformatics data analysis indicates that when ferroptosis is the main type of tumor cell death in glioma patients, it generates an immunosuppressive tumor microenvironment, which contributes to poor therapy outcome [69]. The authors also showed in vitro and in preclinical In vivo studies that ferroptosis promotes polarization of tumor-associated macrophages to an immunosuppressive M2-like phenotype and T-cell exhaustion, reducing anti-tumor activity, whereas inhibition of ferroptosis combined with PD1/L1 immune checkpoint blockade significantly enhances immunotherapy in a GBM xenograft mouse model. In addition, studies have shown that ferroptosis can impair antigen-presenting cells, resulting in reduced anti-tumor immunity and potentially limiting the therapeutic applications of ferroptosis induction [70]. Thus, ferroptosis also promotes an immunosuppressive tumor microenvironment [69], indicating that its inhibition alongside immune checkpoint blockade could also significantly improve therapeutic outcomes in GBM.

Moreover, not all strategies targeting system xc^–^ inhibition or GPX4 suppression to induce ferroptosis are readily adaptable for clinical GBM treatment. For instance, the silencing of system xc^–^ could interfere with glutamate efflux, leading to the development of excitotoxicity, provocation of seizures and neuropathic pain [71, 72]. Although sulfasalazine, an NF-κB and xc^–^ cystine/glutamate antiport inhibitor, demonstrated strong anti-tumor effects In vivo, its phase 1/2 clinical trial (ClinicalTrials.gov: ISRCTN45828668) was prematurely terminated due to a lack of favorable outcomes in the context of malignant glioma progression in adult patients, coupled with severe neurological side effects [73]. Ferroptosis inducers targeting GPX4 too should be considered with caution, as preclinical studies in mice have shown that GPX4 inactivation causes acute renal failure and death in mice [74]. In conclusion, emerging evidence highlights the dual role of ferroptosis in GBM therapy. Future studies will be crucial in elucidating the mechanisms and therapeutic potential of targeting ferroptosis in GBM, and its interplay in the TMZ effects.

## TMZ and pyroptosis

TMZ has been accompanied by pyroptosis (Box 3), an inflammatory form of regulated cell death, that could offer new therapeutic opportunities for GBM treatment (Fig. 4). It has been reported that TMZ can induce pyroptosis in human GBM cell lines (U-87 and U251) and in patients’ GBM tissues [75]. The authors demonstrated that TMZ induction of pyroptosis in GBM cells involved GSDMD. Of note, knockdown experiments revealed that reducing GSDMD levels diminished TMZ-induced pyroptosis and decreased IL-1β expression, underscoring GSDMD’s critical role in mediating this cell death pathway [75]. Interestingly, analysis of public glioma datasets, including the Cancer Genome Atlas (TCGA) (https://portal.gdc.cancer.gov/) and the Chinese Glioma Genome Atlas (CGGA) (http://www.cgga.org.cn/index.jsp), revealed that GSDMD expression is significantly higher in gliomas than in non-tumor brain tissues [75]. Furthermore, its expression is significantly correlated with IDH1/2 mutations, 1p19q co-deletion, and the mesenchymal subtype, suggesting that GSDMD could serve as a potential prognostic biomarker for tumor progression and patient outcomes.

Another research group used TMZ in combination with magnetic temperature-sensitive liposomes (TMZ/Fe-TSL) to induce pyroptosis in human GBM cell lines (U-87 and U251) [76]. While this study demonstrated that abundant ROS generated by TMZ/Fe-TSL under an alternating magnetic field can activate the NLRP3 inflammasome, the authors have not yet explored whether the pyroptosis induced in GBM cells can mediate antitumor immunity [76]. Moreover, an in vitro study has identified miRNA-214 [77] along with its encoded products as potential mediators in the induction of pyroptosis in GBM cells [78]. Furthermore, studies on glioma cohorts from TCGA and CGGA databases revealed that GBM patients with high expression levels of pyroptosis-related genes exhibit glioma progression but show increased sensitivity to TMZ and immunotherapy, including anti-PD1 therapy, and along with enhanced CD8^+^ T cell infiltration [79]. Taken together, these studies suggest that pyroptosis could serve as an effective alternative pathway in novel GBM treatment strategies, by transforming the immunologically “cold” GBM microenvironment and ultimately enhancing the efficacy of GBM immunotherapy. However, research on pyroptosis in GBM remains limited and unclear. Future studies are needed to clarify its role in GBM and its impact on therapeutic outcomes.

## TMZ and cuproptosis

The discovery of a copper-dependent form of regulated cell death by Tsvetkov et al. in 2022 [80] rapidly ignited interest in targeting cuproptosis **(**Box 3**)** as a novel strategy to overcome drug resistance in aggressive tumors. This is especially relevant, as emerging evidence shows that it can be immunogenic, reshaping the tumor microenvironment into an immunologically ‘hot’ state that facilitates the development of a robust antitumor immune response [81, 82].

Recent evidence points to a complex interplay between TMZ and cuproptosis in GBM. in vitro studies have demonstrated that mitochondrial ROS production induced by the copper ionophore elesclomol enhances the sensitivity of GBM stem-like cells and GBM stem-like-derived endothelial cells to TMZ, leading to increased cytotoxicity compared to TMZ alone [83]. The efficacy of this combination was further confirmed In vivo, where a significant reduction in tumor cell proliferation was observed in a mouse brain xenograft model using patient-derived GBM stem-like cells [83]. Significant inhibition of proteasome activity through disulfiram/copper complex application contributed to augmentation of DNA-damaging activity of TMZ in patient-derived GBM stem-like cultures in vitro, and prolonged mouse survival in an intracranial xenograft model established from newly diagnosed as well as recurrent patient-derived GBM tumors [84]. Other recent findings indicate a potential synergistic interaction between cuproptosis and TMZ [85]. Triggering cuproptosis with copper chloride (CuCl_2_) increased TMZ sensitivity in human T98G and U251 GBM cells, reducing their proliferative and migratory capacity. The process was accompanied by accumulation of the copper death marker dihydrolipoamide s-acetyltransferase (DLAT) in mitochondria, culminating in copper-induced cell death [85]. TMZ+CuCl_2_ treatment also significantly suppressed tumor growth without overt signs of systemic toxicity (*i.e*., body weight loss and general behavior) in a murine tumor xenograft model. The antitumor effect was attenuated by overexpression of tripartite motif-containing protein 14 (TRIM14), which led to disruption of the regulatory axis with its downstream effector ATP7A (TRIM14-ATP7A), reducing intracellular copper accumulation, which was associated with poor survival in GBM patients [85].

Studies on the immunogenic potential of combining cuproptosis with TMZ are just beginning to emerge. These primarily nanotechnology-based approaches may also help enhance clinical efficacy, especially as the first clinical efforts to modulate copper homeostasis to enhance TMZ efficacy have shown extended remission only in a limited number of patients with BRAF-mutant GBM [86] or failed to significantly improve six-month overall survival (ClinicalTrials.gov: NCT02678975) [87]. In this context, an ion-sensitive hydrogel platform (tdIHG) was recently devised for the nose-to-brain co-delivery of TMZ and disulfiram to treat GBM, with additional oral copper supplementation [88]. in vitro studies using human glioblastoma U251 and rat glioma C6 cell lines demonstrated that a combination of copper and tdIHG increased cellular sensitivity to TMZ, and significantly increased DAMPs secretion (*i.e*., HMGB1 and ATP secretion and CRT exposure) and stimulated CXCL10 release. In a rat orthotopic GBM model, tdIHG+Cu treatment prolonged median survival, effectively reduced tumor growth, and exhibited a high safety profile [88]. Notably, this therapeutic effect was accompanied by a significant increase in dendritic cell maturation in tumor-draining lymph nodes, and stimulated CD8 + T cell infiltration into the tumor tissue, ensuring the formation of a robust antitumor immune response [88]. A novel albumin-based nanodelivery system (BSA@CTW-NPs) was developed for the co-delivery of TMZ and Wogonoside [89]. BSA@CTW-NPs triggered the generation of a significant amount of reactive ROS in the tumor microenvironment and simultaneously provided the excess copper ions in GBM cells to trigger cuproptosis. This treatment facilitated the exposure of three key DAMPs (*i.e*., ATP, CRT and HMGB1) from murine GL261 cells and promoted the maturation of bone marrow-derived DCs co-cultured with the BSA@CTW-NPs-treated GBM cells. Integration of BSA@CTW-NPs in hemostatic sponges for postoperative tumor cavity tamponade in immunocompetent C57BL/6 mice significantly suppressed tumor recurrence by remodeling the tumor microenvironment. This effect was achieved through the activation of DC-mediated antigen presentation, enhancement of cytotoxic CD8 + T cells, attenuation of immunosuppressive Treg populations, and suppression of M2 macrophage polarization [89].

In summary, current findings indicate that copper ionophores could synergize with TMZ to overcome resistance and enhance therapeutic efficacy in GBM. However, further mechanistic and translational studies are required to determine whether cuproptosis meets the canonical criteria of immunogenic cell death and to define its potential role in reshaping the GBM tumor microenvironment and generating long-lasting anti-tumor immunity.

## Conclusion and future perspectives

GBM is the deadliest malignant primary brain tumor. The current FDA-approved standard of care for GBM includes maximal safe surgical resection of the tumor, followed by RT and TMZ chemotherapy [5, 90]. This conventional therapy aims to damage the DNA in residual tumor cells after surgery using RT, which reduces the risk of rapid regrowth and facilitates the action of TMZ as an alkylating agent to eliminate tumor cells further and help delay postoperative recurrence. Moreover, specific RT regimens can induce ICD, releasing tumor-associated antigens and DAMPs that stimulate DC activation and T-cell priming [91]. The role of preoperative RT in reshaping the immunologically ‘cold’ tumor microenvironment and improving conventional radio-chemotherapy has also emerged in preclinical studies and early-phase clinical trials [7] (ClinicalTrials.gov: NCT05030298, NCT03582514). Meanwhile, TMZ reduces immunosuppressive regulatory T cells and myeloid-derived suppressor cells, promoting a more favorable immune milieu. However, GBM’s inherently immunosuppressive microenvironment, characterized by low tumor mutational burden and limited T-cell infiltration, often limits the durability of these responses.

Additionally, the heterogeneity of GBM cells and their prominent plasticity frequently leads to resistance against TMZ treatment, significantly diminishing its clinical benefit. Achilles’ heel in GBM sensitivity to TMZ has been primarily attributed to genetic factors, such as the MGMT expression level [25] and functional DNA mismatch repair [92]. The list of potential mechanisms of TMZ resistance has recently been expanded to include several epigenetic mechanisms [31, 32], and this list is still growing. Notwithstanding these challenges, numerous advantages, including the predictable and reversible TMZ side effects and the ability to cross the blood-brain barrier, maintain TMZ as the cornerstone chemotherapeutic agent for GBM treatment and ignite a keen interest in pursuing breakthrough strategies for overcoming TMZ resistance in GBM.

GBM requires a multimodal approach to treatment with a focus on a personalized therapeutic regimen, but the presence of immunotherapeutic approaches in this scheme is crucial for eliminating any remaining tumor cells and decreasing the risk of tumor recurrence. Interestingly, TMZ can enhance immunotherapy outcomes. Although the action of TMZ is closely related to the development of lymphopenia [93], TMZ can play an immunomodulatory role in synergy with GBM immunotherapy. Preclinical studies and clinical trials have suggested rapid expansion of antigen-specific T cells during homeostatic lymphocyte recovery [94]. The timing and dosing of TMZ regimens can also significantly alter the amount of regulatory T cells and has great potential to influence the function of DCs [94].

Recent studies have provided additional insights into the immunogenic effects of RT in GBM treatment. These could be employed as an additional asset in this battle. However, the timing and dosing of RT are critical, as suboptimal regimens may fail to induce sufficient ICD [95]. Beyond its direct cytotoxic effects, RT can also as a modulator of the tumor microenvironment to enhance immunogenicity, thus providing a rationale for integrating these approaches with immunotherapy strategies [96]. Immune checkpoint blockade may enhance the immunogenic effects of RT and TMZ, though further studies are needed to identify predictive biomarkers and optimize treatment protocols [97]. In this regard, there is a need for extensive exploration of the role of the tumor microenvironment in mediating resistance to RT and TMZ. A recent study highlights the role of a complex interplay between tumor-associated macrophages, microglia, and T cells in shaping the immune response to GBM [98]. These and other findings may form the basis of future studies and clinical trials aimed at modifying and improving treatments to limit suppression of the antineoplastic immune response based on changes measured in real time in patients.

Among the immunotherapeutic strategies being developed for GBM treatment in combination with TMZ-based radio-chemotherapy, ICD induction approaches (Box 3) have recently been proposed to enhance the therapeutic response [16]. Here, we provide evidence that there is a favorable interaction of TMZ with regulated cell death modalities associated with ICD, such as necroptosis, ferroptosis, pyroptosis, and the newly emerged cuproptosis with prospective immunogenic potential (Fig. 4). Looking ahead, we propose a novel anti-GBM strategy combining TMZ-induced cytotoxicity with physical or pharmacological ICD inducers, potentially producing mixed-type cell death and offering new opportunities to enhance GBM treatment efficacy [16]. Switching from TMZ-based cell death mechanisms to an alternative cell death pathway could enhance the sensitivity of heterogeneous GBM cell populations, reducing the risk of adaptive resistance, and ultimately overcoming GBM cell resistance, which arises when therapy targets a single cell death pathway [16]. Additionally, triggering ICD modalities with ICD-promoting agents may also contribute to the generation of an immunologically favorable GBM microenvironment [10, 15] and amplify the immune system response by activating ICD-associated anti-tumor immunity and establishing long-lasting immunological memory.

Herein, we highlight the diversity of ICD-promoting agents, including ferroptosis inducers, necroptosis-sensitizing strategies, pyroptosis activators, and emerging metabolic interventions such as copper ionophores. When these agents are combined with TMZ, they exhibit enhanced efficacy against GBM. Accordingly, we propose that the selection of the most appropriate ICD agent to augment TMZ-based therapy should follow a patient-specific approach. The potential intrinsic risk of resistance of a patient’s GBM to the intended regulated cell death modality should first be assessed. This can be facilitated by the development of bioinformatics-based analytical tools and prognostic risk models. Moreover, the design of mutually beneficial activation of TMZ and ICD modalities should be finely tuned, as failure to maintain the balance between the types of cell death in mixed-type of cell death could potentially lead to the development of a pro-tumorigenic effect and abrogate the therapeutic efficacy [71, 73, 74, 79]. Therefore, current research efforts should prioritize investigating the interplay between regulated cell death pathways and the key mechanisms involved in TMZ activity, specifically apoptosis and autophagy [99, 100]. Cross-talk among different cell death modalities, particularly apoptosis, ferroptosis and autophagy, has been observed [99, 101] and many novel insightful studies are anticipated.

Maintenance of a balance between TMZ and ICD inducers, including dosage control and yield rates, can be achieved by using the nanomedicine approach [102, 103]. For example, novel designs for the targeted delivery of TMZ to GBM cells *via* nanocarriers are currently being developed [104, 105]. More importantly, the design of nanocarriers offers the option of impregnating them with other compounds, including ICD inducers or other immunologic adjuvants, which can help achieve multiple therapeutic goals.

Evaluation of the safety and biological efficacy of the developed strategies requires both in vitro *and* In vivo experimentation. These studies should preferably be based not only on preclinical standards that access general and specific toxicity effects, but also on the “gold” standards for evaluation of ICD in oncological models and anti-cancer vaccination strategies [106, 107]. We also emphasize that in the strategy being developed, it is necessary to use TMZ doses, solvents, and modes of exposure that are closest to clinical settings; this condition is often overlooked in experimental studies, where a single high dose of TMZ is used instead of multiple doses, or DMSO is used as a solvent [108].

In conclusion, GBM treatment requires combinatorial strategies together with immunotherapeutic procedures. Each therapy must be carefully tailored with personalized dosages and treatment regimens. Further detailed research into the mechanisms of TMZ resistance and identifying beneficial and balanced interactions between ICD modalities could facilitate the development of breakthrough approaches for enhancing GBM therapy.
